# Bone mineral density 5 to 11 years after metabolic and bariatric surgery in adolescents with severe obesity compared to peers

**DOI:** 10.21203/rs.3.rs-3345103/v1

**Published:** 2023-09-21

**Authors:** Halley Wasserman, Todd Jenkins, Thomas Inge, Justin Ryder, Marc Michalsky, Stephanie Sisley, Changchun Xie, Heidi Kalkwarf

**Affiliations:** Cincinnati Children’s Hospital Medical Center; Cincinnati Children’s Hospital Medical Center; Lurie Children’s Hospital of Chicago; Lurie Children’s Hospital; Center for Healthy Weight and Nutrition at Nationwide Children’s Hospital, Columbus, Ohio; Baylor College of Medicine; Cincinnati Children’s Hospital Medical Center, Cincinatti OH

**Keywords:** bone mineral density, bariatric surgery, adolescent, obesity

## Abstract

**Objective:**

Metabolic and bariatric surgery (MBS) is associated with decreased bone mineral density (BMD) in adults. The long-term impact of MBS during adolescence on BMD is unknown. We report bone health status 5 to 11 years after Roux-en-Y gastric bypass (RYGB) and vertical sleeve gastrectomy (VSG) from the Teen-LABS study cohort.

**Methods:**

Between 2016 and 2022, BMD was measured by dual energy x-ray absorptiometry (DXA) in 106 young adults who had undergone MBS as adolescents. Volumetric BMD by peripheral quantitative computed tomography was measured on a subset. Ninety-one controls who had not undergone MBS were recruited for comparison.

**Results:**

Compared to controls, adjusted mean DXA-BMD of the RYGB (n = 58) and VSG (n = 48) groups were lower at the hip (−10.0% and − 6.3%), femoral neck (−9.6% and − 5.7%) and ultra-distal radius (−7.9% and − 7.0%; all *p* < 0.001), respectively. DXA-BMD did not differ between RYGB and VSG groups. Trabecular volumetric BMD at the radius and tibia were lower in the RYGB (−30% and − 26%) and VSG (−15% and − 14%) groups compared to the control group (*p* < 0.001). Greater time since MBS was associated with lower BMD Z-scores at the hip (*p* = 0.05) and femoral neck (*p* = 0.045). Percent change in body mass index (BMI) from baseline or in the first year after MSB were not associated with bone measures at a median of 9.3 years post MSB.

**Conclusion:**

BMD, especially of the hip and femoral neck, was lower in young adults who underwent MBS during adolescence compared to matched peers who had not undergone MBS. BMD Z-scores of the femoral neck decreased with time since MBS but were not associated with BMI change.

## INTRODUCTION

Metabolic and bariatric surgery (MBS) has beneficial effects on obesity-related cardiometabolic comorbidities and risk factors in both adolescents and adults (1–5), however, few studies address concerns for impaired bone health. Following Roux-en-Y gastric bypass (RYGB) in adults, declines in dual x-ray absorptiometry (DXA) acquired areal bone mineral density (BMD) at the hip and/or lumbar spine have been reported (6–8). The magnitude of weight loss in the first year following RYGB has been associated with the decrease in BMD one year post-procedure (8) though this association was not evident two years post-procedure (7). Recent reports show that declines in BMD continue five years post-MBS and beyond (9, 10) when weight loss has plateaued. Changes in markers of calcium homeostasis and bone turnover have been found in the first six months after MBS and turnover markers remain elevated (11), with the elevation being greater in adults who underwent RYGB compared to those who underwent vertical sleeve gastrectomy (VSG) (12). Intestinal calcium absorption was reduced 6 months after RYGB and VSG in adults (13, 14), and parathyroid hormone (PTH) levels were persistently elevated five years later (15, 16). These metabolic changes following MBS may explain part of the persistent decline in BMD up to 4 years following MBS and may contribute to the increased incidence of overall fracture in adults 3 years after MBS compared to controls of similar age, sex and clinical health (17). Risk of major osteoporotic fractures is increased 3 to 5 years post-surgery in some (18–20) but not all (21) adult studies. The 20-year cumulative risk of fracture increased 2.5-fold in those who underwent RYGB as adults whereas risk was not significantly increased in those who underwent VSG (22).

For adolescents who undergo MBS during the time of peak bone accrual, the long-term consequences of bone loss for fracture risk may be even greater. BMD outcomes following MBS in adolescents is limited. In studies reporting a maximum follow up duration of 2 years, RYGB has been associated with significant declines in WB BMD (23–25) whereas VSG was associated with significant declines in hip BMD (26, 27). Similar to findings in adults, c-terminal telopeptide and PTH increased following MBS in adolescents (24, 28, 29). Currently, only one study has reported fracture outcomes in adolescents post-MBS, and no fractures were reported 2 years post-MSB (24). If bone outcomes following adolescent MBS follow a trajectory similar to adults, even small annual declines in BMD may result in significant bone deficits over time.

The Teen-Longitudinal Assessment of Bariatric Surgery (Teen-LABS) study followed individuals who underwent MBS as adolescents. To assess the long-term impact on bone health of MBS performed during adolescence, we report BMD and related outcomes in those former adolescents who were median 9.3 years post-MBS compared to a non-surgical control group frequency matched on body mass index (BMI), age, race, and sex. We hypothesized that BMD at the total hip (TH) and femoral neck (FN) would be lower in participants who underwent RYGB or VSG during adolescence compared to controls. We performed secondary analyses to assess whether BMD varied by surgery type (RYGB vs. VSG), time since surgery, and weight loss after surgery, and whether fracture rates differed among surgical and control groups.

## METHODS

### Study Participants

Teen-LABS (NCT00465829), a multicenter, prospective observational cohort study of the risks and benefits of MBS, consecutively enrolled 271 adolescents (ages 13 to < 19 years), who underwent either RYGB, VSG or laparoscopic adjustable gastric band (LAGB) between March 2007–February 2015 at 5 U.S. centers. Study details have been previously described (30). Teen-LABS participants from the Cincinnati Children’s Hospital (CCHMC, Cincinnati, OH), Nationwide Children’s Hospital (NCH, Columbus, OH) and Texas Children’s Hospital (Houston, TX) centers were eligible to participate in the bone health study when they were 5 to 11 years post-MBS. The other two Teen-LABS centers did not have access to the same type of densitometer. For this long-term bone health study, enrollment was limited to Teen-LABS participants who underwent RYGB or VSG but not LAGB due to its low use within the overall cohort (n = 14). To strengthen statistical power for comparisons between RYGB and VSG, we recruited additional participants (n = 32) who underwent VSG as adolescents between March 2012 to November 2015 at CCHMC and NCH. Additionally, we recruited a non-surgical control group from the three participating centers whose age, sex, race and BMI distributions were similar to surgical participants. Exclusion criteria were non-ambulatory status, genetic bone disorders, primary hyperparathyroidism, hyperthyroidism, Cushing syndrome, hypogonadotropic hypogonadism, syndromic obesity or use of medications known to appreciably affect bone density; history of chemotherapy or radiation treatment; pregnancy within 3 months; lactating within 6 months; Depo-Provera contraceptive use within 3 years, or cumulative use greater than 24 months; and weight exceeding 181.8 kg. The Institutional Review Boards of each center approved the study protocol, and participants provided informed consent.

### Bone Densitometry

We prospectively obtained DXA scans of the whole-body (WB), lumbar spine (LS), left hip and non-dominant forearm with use of Hologic (Marlborough, MA) densitometers. Body regions with indwelling hardware were not scanned. Calibration stability was assessed daily by scanning a machine-specific spine phantom. Participants were positioned according to manufacture guidelines. All scans were analyzed centrally at CCHMC using software version 13.5.3.1 to quantify BMD (g/cm^2^) of the WB, LS, TH, FN, ultra-distal radius, and 1/3rd radius. Age-, sex- and race-specific BMD Z-scores were generated by the Hologic software using reference data from National Health and Nutrition Examination Survey (NHANES) for the WB, TH and FN, and from a Hologic derived sample for the LS and radius.

Peripheral quantitative computerized tomography (pQCT) scans were obtained on participants at CCHMC. Scans were acquired using the Stratec XCT 2000 scanner (Stratec Medizintechnik, GmbH), with a voxel size of 0.4 mm and a speed of 25 mm/sec. Scans were acquired of the tibia and radius at the distal 4% and 30% sites. Tibia scans were not obtained for participants with metal hardware or leg size too large for the pQCT gantry. The 4% sites are within the distal metaphysis and are composed primarily of trabecular bone. The 30% sites are in the diaphysis and are predominantly cortical bone. Scans were analyzed using the manufacturer’s software version 6.0 to yield measures of trabecular and cortical volumetric density (vBMD, mg/cm^3^), cortical thickness (mm), periosteal circumference (mm), and strength-strain index (SSI, mm^3^). SSI is a biomechanical measure reflecting the strength of bone to withstand torsion and bending forces. pQCT calibration stability was assessed by scanning a phantom and evaluating the vBMD results prior to participant scanning.

All DXA and pQCT scans were visually assessed for movement, positioning, artifacts, and image degradation due to excess soft tissue. Scans deemed invalid were not used. Among DXA scans, 5 WB, 8 LS, 7 TH / FN, 2 ultra-distal radius and 3 1/3 radius scans were considered invalid. Among pQCT scans, 1 scan was considered invalid at each of the following sites: 4% radius, 30% radius and 4% tibia.

### Potential Covariates

Height and weight were measured in triplicate with participants dressed in light clothes without shoes. Participants completed the Block calcium vitamin D screener (NutritionQuest, Berkeley, CA), which queried intake of 19 food items, supplements, and fortified foods and yielded information on total calcium and vitamin D intakes from dietary sources plus supplements.

Participants completed International Physical Activity Questionnaire (IPAQ) - long version. IPAQ asks about time performing moderate or vigorous activities in the last 7 days for five domains: job-related; transportation; housework, house maintenance and family care; recreation, sports and leisure; and sitting. Using the IPAQ scoring methodology, physical activity data are presented as metabolic equivalent (MET)-minutes/week.(31)

Participants completed questionnaires regarding medical conditions, current medications, menstrual frequency in the last year, and tobacco use. We classified participants as having diabetes if they had a diagnosis of diabetes and were currently using insulin or oral medications. We tabulated the frequency of medications reported to be associated with bone density, i.e., selective serotonin reuptake inhibitors (SSRI) (32), proton pump inhibitors (PPIs) (33) and inhaled steroids (34).

Participants reported the number of prior fractures and age at fracture. We classified fractures as before or after MBS. Non-fasting blood samples were obtained on participants who underwent MSB. Samples were analyzed for 25-hydroxyvitamin D and PTH at the Northwest Lipid Metabolism and Diabetes Research Laboratories (NLMDRL, n = 86) or Quest Diagnostics (n = 12). Both sites used an intact PTH immunoassay. The NLMDRL used HPLC-tandem mass spectrometry for measurement of 25-hydroxyvitamin D whereas Quest Diagnostics used an immunoassay.

### Statistical Analyses

We examined the distribution of all variables, and non-normally distributed variables were transformed to better approximate a normal distribution. Continuous variables were summarized as means ± SD or median and inter-quartile ranges (IQR), and categorical variables were summarized as counts and percentages. Group characteristics were compared by t-test or ANOVA for continuous variables and by Chi-square or Fisher’s exact test for categorical variables.

Regression modeling was used to test for differences in bone outcomes among groups. Analyses were conducted first without then with adjustment for covariates. Covariates included in adjusted models for all outcomes regardless of statistical significance were clinical center, height (DXA outcomes) or limb length (pQCT outcomes), BMI, sex, and black race to reduce variability and potential confounding. We also tested covariates that differed among groups (*p* < 0.10) and kept covariates that were associated with the bone outcome at *p* < 0.10. Group differences were considered statistically significant with a *p*-value < 0.05.

We tested whether bone density differed between RYGB and VSG groups and the influence of time since surgery on attained BMD. These analyses were restricted to bone outcomes that significantly differed among surgical and control groups (*p* < 0.05). Because time since surgery was related to surgery type (Table 1), we tested both in the same model. We also examined whether bone density was associated with percent change in BMI since surgery and in the first year after surgery. For these analyses, we included covariates that were retained in the models developed above. We compared the frequency of fracture among groups, however, given the small sample sizes, these analyses were considered exploratory.

## RESULTS

Of the 213 participants actively enrolled in the main Teen-LABS study between 2016 and 2022 at one of the 3 bone health study centers, 95 declined participation and 12 did not meet eligibility criteria. The final sample included 106 participants: 58 had undergone RYGB and 48 VSG. Although not systematically ascertained, common reasons for non-participation were skipping in-person visits due to distance from their surgical center where the DXA machines were located and COVID-19 related restrictions on research operations. Teen-LABS participants enrolled in the study were similar to those who chose not to enroll in terms of age at MBS, sex, race, Hispanic ethnicity, and baseline BMI (data not shown). However, Teen-LABS participants that had bone measurements experienced a greater mean percent reduction in BMI at 5 years following MBS than those who did not participate (−26% vs. −18%). At the same 3 centers, 91 individuals who had not undergone MBS (controls) were enrolled. Control and MBS groups were similar in terms of sex, race, Hispanic ethnicity, prevalence of diabetes, physical activity, calcium intake and use of SSRIs, PPIs and inhaled steroids (Table 1). At bone study enrollment, the VSG group was slightly younger and had a lower BMI compared to the RYGB and control groups. The time since MBS was also shorter for the VSG group vs. RYGB group, but there was no difference in percent weight loss since surgery or in the first year after surgery. The control group had lower vitamin D intake and were less likely to smoke cigarettes than the surgical groups. Because vitamin D intake was inversely associated with bone density, we did not include it in multivariable models as it likely reflected confounding by indication.

Among participants who had undergone MSB, vitamin D status reflected by serum 25-hydroxy vitamin D concentration was poor, and almost half were vitamin D deficient (25-hydroxy vitamin D < 20 ng/mL). There was no significant difference in median serum 25-hydroxy vitamin D between RYGB and VSG groups (Table 1). In contrast, PTH concentrations were higher (p < 0.001) in individuals who had undergone RYGB compared to those who had undergone VSG (Table 1). These findings persisted and the magnitude was not altered when controlling for laboratory site (data not shown).

### DXA

Unadjusted mean BMD Z-scores for all groups were ≥ 0.0 at all skeletal sites except the WB for which mean Z-scores were statistically < 0.0 for all groups (Table 2). Unadjusted mean BMD and BMD Z-scores were lower at the TH, FN and ultra-distal radius in RYGB and VSG groups than the control group, but significant differences were not detected between surgical groups.

**Figure 1:**
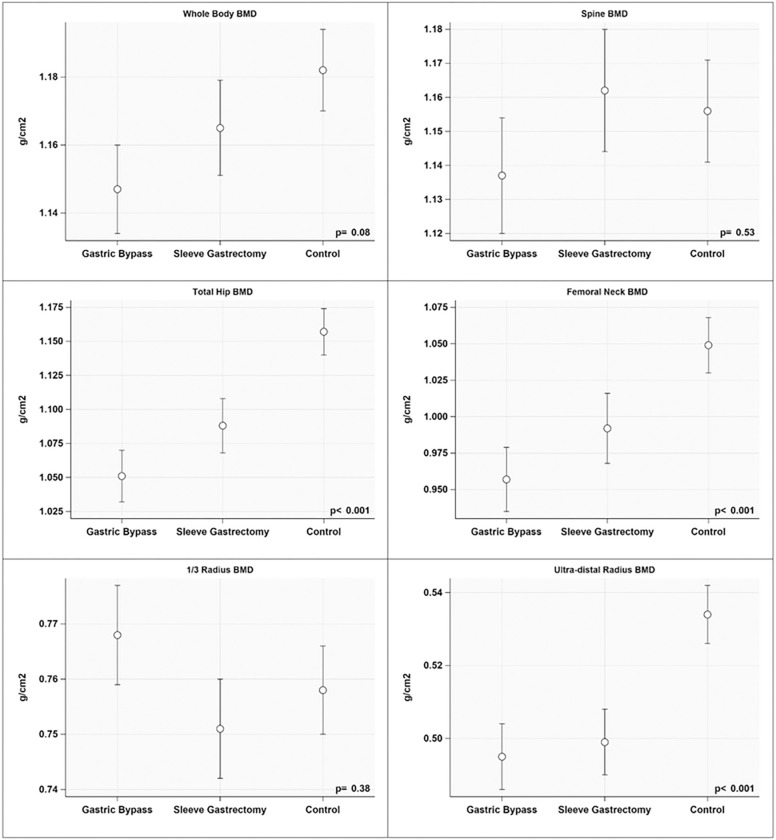
Adjusted mean dual energy x-ray absorptiometry (DXA) outcomes for metabolic and bariatric surgery groups and matched controls. Covariates included in regression models for all bone outcomes regardless of statistical significance were clinical center, height, body mass index, sex, and black race. Femoral neck bone mineral density (BMD) regression model also included age. RYGB = Roux-en-Y gastric bypass; VSG = vertical sleeve gastrectomy

When controlling for covariates, BMD of the RYGB and VSG groups remained lower than controls at the TH (−10.0% and − 6.3%), FN (−9.6% and − 5.7%) and ultra-distal radius (−7.9% and − 7.0%; Fig. 1). Adjusted mean BMD at the WB, LS and 1/3rd radius did not differ significantly among groups.

We investigated the relation between surgical group and time since surgery with BMD at the FN, TH, and ultra-distal radius in participants who had undergone MBS. There was no significant difference in BMD between RYGB and VSG groups when controlling for time since surgery for any bone outcome (all *p* ≥ 0.4) (**Supplemental Table 2**). However, time since surgery was inversely associated with FN and TH BMD Z-scores (*p* ≤ 0.05); Z-scores were − 0.18 ± 0.9 and − 0.16 ± 0.08 lower, respectively, for each additional year after MBS. The inverse associations between time since surgery and ultra-distal radius BMD and BMD Z-score did not meet statistical criteria (*p* > 0.05). There was no significant association between BMD or BMD Z-scores of the FN, TH or ultra-distal radius and percent change in BMI since MBS or in the first year after surgery (all *p* > 0.05).

### Peripheral QCT

**Figure 2:**
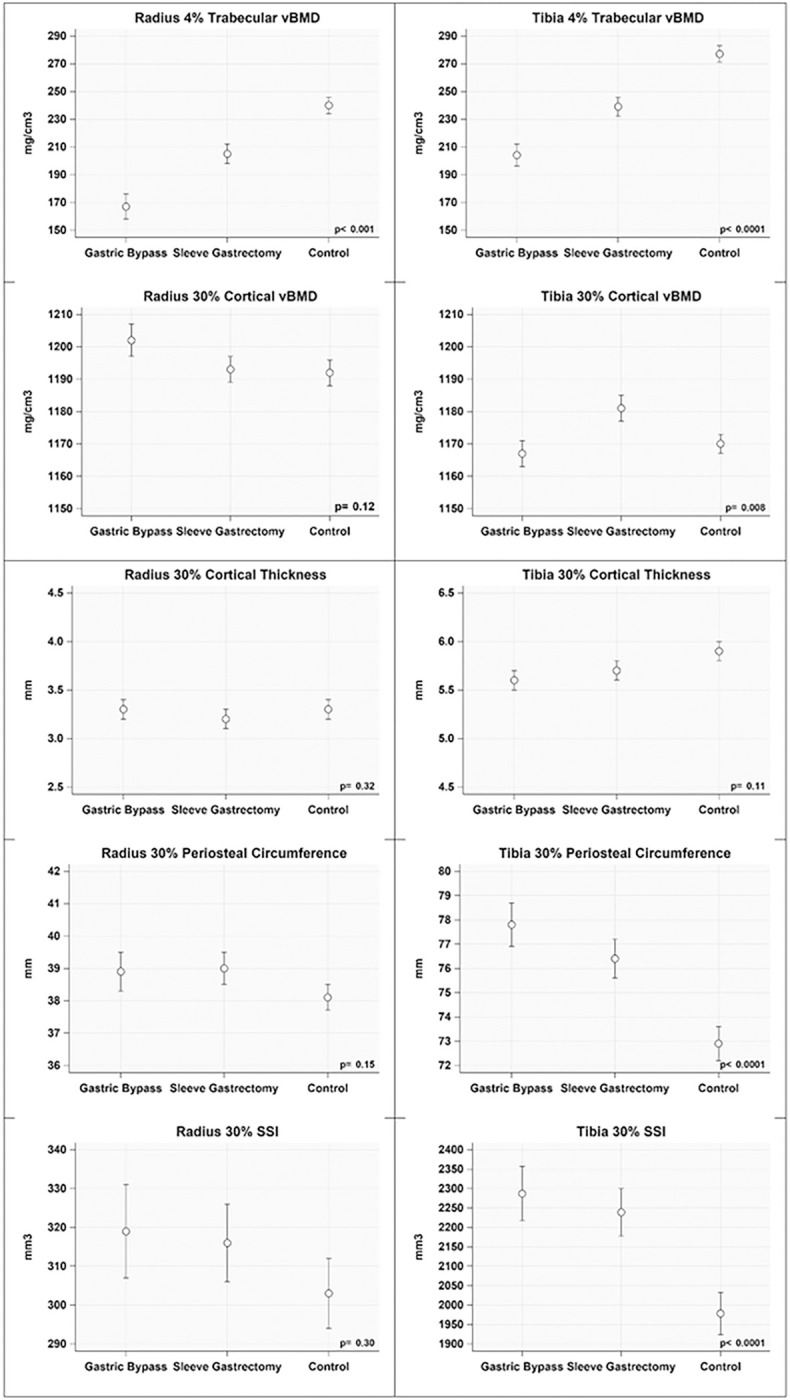
Adjusted mean peripheral quantitative computed tomography (pQCT) outcomes for metabolic and bariatric surgery groups and matched controls. Covariates included in regression models for all bone outcomes regardless of statistical significance were clinical center, limb length, body mass index, sex, and black race. Measurements were obtained of the distal radius and distal tibia at sites 4% and 30% from the distal endplates.

Trabecular vBMD at the distal 4% radius and 4% tibia sites measured by pQCT were significantly lower in RYGB and VSG groups compared to controls in both crude (Supplemental Table 1) and covariate adjusted analyses (Fig. 2). Group differences in cortical vBMD were evident at the 30% tibia but not 30% radius. There were no significant differences among groups in cortical thickness of either the radius or tibia. Periosteal circumferences of both were greater in the MSB groups than in controls. SSI at the 30% tibia was greater in the MSB groups than in the control group, potentially due to the greater periosteal circumference.

Trabecular vBMD was significantly lower in the RYGB group than in the VSG group at the radius and tibia, −13.3% (*p* = 0.04) and − 13.4% (*p* = 0.03), respectively. Time since surgery was not associated with trabecular vBMD at the 4% radius and tibia sites (**Supplemental Table 2**). Percent change in BMI since surgery or in the first year after surgery were not associated with total or trabecular vBMD at either the 4% radius or tibia sites (all *p* > 0.3, data not shown).

### Fractures

There was no significant difference (*p* = 0.22) in occurrence of ever sustaining a fracture among groups. The number of participants who ever sustained a fracture was 15/58 (34%) in the RYGB group, 19/48 (53%) in the VSG group and 40/91 (46%) in the control group. Among those who had undergone MSB and recalled timing of their fracture, 13/13 (100%) in the RYGB group and 15/18 (83%) in the VSG group fractured after MBS.

## DISCUSSION

At a median duration of 9.3 years after adolescent MBS, young adults had lower BMD at both weight bearing (TH, FN) and non-weight bearing (distal radius) skeletal sites when compared to young adults of similar age, sex, race and BMI who had not undergone MBS. While DXA BMD Z-scores at the FN and TH did not differ between RYGB vs. VSG participants, BMD Z-scores were lower in those who were farther out from either procedure, indicating a need for ongoing monitoring of skeletal health following adolescent MBS. Percent change in BMI since MBS and in the first year after MBS were not associated with any skeletal outcomes in this cohort, raising the possibility that additional mechanisms affect attained BMD after MBS.

Our findings at the hip were similar to those reported by Nimmala *et al*. who found that BMD Z-scores at the TH and FN significantly decreased over 12 months in adolescents who underwent VSG relative to non-surgical controls, but no significant change was noted at the WB (28). Mitchell *et al*. followed this cohort for another 12 months (27) and noted a reduction from baseline in FN BMD by 8.9% and TH BMD by 8.4% at 24 months post-surgery but found no significant change at the WB or 1/3 radius. However, in both studies, differences in BMD between VSG and non-surgical control groups were no longer significant after controlling for change in BMI. In contrast, we did not find that change in BMI since surgery or in the first year after surgery were associated with attained BMD a median of 9.3 years after MSB. The longer time since MSB in our study may have allowed opportunity for other factors to affect bone density. Also, we could not estimate loss of BMD in our cross-sectional study, preventing a direct comparison of findings. Our focus was BMD after adaptation to weight change compared to weight similar controls to assess the ability of bone to adapt to prevailing weight bearing forces.

BMD of trabecular and cortical bone by pQCT were consistent with what was observed with DXA. vBMD of trabecular bone at the 4% distal radius and tibia by pQCT were significantly lower in persons who had undergone MBS compared to those who had not, but there was no difference at the 30% distal radius by pQCT reflecting cortical bone. Using high resolution-pQCT (HR-pQCT), Nimmala *et al*. (28) and Mitchell *et al*. (27) also found that trabecular vBMD at the distal radius and distal tibia decreased over 12 months and 24 months in persons who underwent adolescent VSG. Though surgical type did not affect DXA BMD outcomes in our study, trabecular vBMD measured by pQCT was lower in RYGB vs. VSG recipients. PTH was higher in persons who had undergone RYGB than those who had undergone VSG, similar to results from adult MBS patients (11, 35), which may explain trabecular vBMD differences.

The clinical implications of lower trabecular bone vBMD at the distal tibia and radius in young adults who underwent adolescent MBS are unclear. Adults who undergo MBS have a greater risk of major fracture (including hip and distal radius) that increases over time: compared to incident fracture rates 5 years prior to MBS, the incidence rate ratio was 2.77 at < 3.0 years post-surgery and 3.78 at 3.1–5.0 years post-surgery (20). The increased fracture risk was associated with RYGB as opposed to VSG, but data are limited to small number of participants and potentially biased study designs (36, 37). In our cohort, there was no difference in the prevalence of ever experiencing a fracture between persons who had undergone MSB approximately 9 years prior and those that did not. Interestingly, persons who had undergone MBS were more likely to fracture after MSB than before. This may have been a consequence of bone loss after surgery, or it could have reflected increased physical activity after weight loss as physical activity is a predictor of fracture among youth (38, 39), though there were no group differences in reported physical activity. Participants in this study were relatively young and at low risk of hip and vertebral fracture. The greater periosteal circumference at the distal tibia in surgical vs. control groups may be a protective mechanism dispersing loading force on the long bone in the event of a fall.

Trabecular bone occupies most of the skeletal area within the vertebra. While no difference in DXA LS BMD between surgical vs. control groups was noted in our cohort, Huber et al reported declines in LS trabecular vBMD and vertebral body strength using quantitative CT at 24 months following adolescent VSG compared to non-surgical controls (40). This finding may explain the adjusted relative risk of 1.70 for clinical spine fractures following MBS in adults compared to non-obese controls (18). Further research to determine the impact of adolescent MBS on spinal bone health is necessary.

We first reported concerns about impaired bone health following adolescent RYGB in 2011 (23) as we found that WB BMD Z-scores decreased by nearly 1 SD in the first 12 months following surgery with continued though smaller declines between 12 to 24 months after surgery (23, 24). At all timepoints, mean WB BMD Z-scores remained above 0.0. BMI-matched controls were not included. In our current study, despite the lower BMD at the TH, FN, and distal radius in those who underwent MSB compared to controls, the BMD Z-scores for all skeletal sites except the WB were also at or above the median for their age, sex, and race. BMD is well known to increase with BMI as the skeleton adapts to support a greater body weight. It is not clear why WB BMD Z-scores in our current study were below 0.0, especially in the control group. Interestingly, the WB BMD Z-scores were also below 0.0 in the study by Nimmala *et al*. Percent body fat and visceral adiposity have been inversely associated with WB BMD in adults (41, 42) and adolescents (43, 44). Calibration of DXA machines used in this study and those used to generate the reference ranges should be considered as intermachine calibration differences are greater for the WB scans than for TH and LS scans (45).

About 50% of individuals were deficient in vitamin D at a median of 9.3 years after MBS, similar to what we found in the Teen-LABS cohort 5 years post-MBS (29). The prevalence of vitamin D deficiency was potentially even higher in controls whose vitamin D intake was lower than in MSB cases. We previously reported that the prevalence of elevated PTH rose from baseline to 5 years post-MBS among the RYGB group (29). These findings parallel that found in an adult cohort and suggest that RYGB may be associated with chronic disruptions of calcium homeostasis (15). PTH effects on bone turnover are complex. Intermittent increases in PTH stimulate bone formation, whereas sustained high levels of PTH lead to bone resorption (46). Hypocalcemia is the key stimulus of PTH release. In our study, estimated calcium intake was insufficient in all participants and thus would not explain group differences in BMD. We do not have measures of intestinal calcium absorption or urinary excretion, which would affect prevailing circulating calcium levels and calcium balance. Nonetheless, calcium intake is an important risk factor to address post-MBS.

Our study is limited in that presurgical BMD measurements were not obtained so we could not calculate bone loss. However, we compared the BMD of those with exposure to MBS in adolescence to a control group without such exposure but were of similar BMI. Mean BMD Z-scores were still within normal limits in all groups. However, we estimated a decrease in BMD Z-scores of 0.18 SD per year at the FN. If this rate continues over time, these young adults may have low BMD before age 50. This cohort underwent MBS after the completion of puberty (Tanner Stage IV-V and skeletal maturation of at least 95%). The 2018 American Society for Metabolic and Bariatric Surgery Pediatric Committee recommended removing this requirement for MBS (47). MBS in pre- and early pubertal children, who have not yet experienced peak bone accrual velocity, may have even greater effects on bone density.

In conclusion, young adults a median of 9.3 years after MBS as adolescents, bone density, especially at the FN, was lower than in young adults of similar BMI who had not undergone MSB. BMD Z-scores at the FN decreased with time since MSB and occurred independent of surgical type and percent weight loss. The clinical impact of BMD changes on osteoporotic fracture risk may not be fully appreciated until sixth decade of life.

## Figures and Tables

**Table 1.** Characteristics of participants in the Teen-LABS bone density study.

Measure	MBS cases		Controls	*P*-value
	RYGB	VSG			
N	58	48	91	
Time since surgery (y)	10.0 (1.0)	7.7 (1.5)	-	<0.001
5.0 – 7.9 years, n (%)	2 (3%)	27 (56%)	-	<0.001
8.0 – 9.9 years, n (%)	16 (28%)	16 (33%)	-	
10.0 – 11.5 years, n (%)	40 (69%)	5 (10%)	-	
Age (y)	26.8 (1.9)	25.1 (2.1)	26.5 (2.7)	<0.001
Female, n (%)	44 (76%)	31 (65%)	73 (80%)	0.13
African ancestry, n (%)	15 (26%)	10 (21%)	16 (18%)	0.48
Hispanic ethnicity, n (%)	5 (9%)	1 (2%)	5 (6%)	0.40
Height (cm)	168.7 (8.8)	169.5 (9.0)	169.2 (8.9)	0.88
BMI current (kg/m^2^)	42.1 (9.9)	37.1 (8.4)	40.2 (8.7)	0.02
BMI pre-surgery (kg/m^2^)	54.5 (9.5)	49.2 (6.1)	-	0.001
BMI change from baseline (%)	−21.6 (17.7)	−24.6 (14.2)	-	0.35
BMI change baseline to 1 y post-surgery (%)	−30.9 (8.8)	−31.8 (8.9)	-	0.63
Total Calcium intake (mg/d)	474 (341, 865)	587 (350, 880)	431 (302, 598)	0.09
Total Vitamin D intake (IU/d)	410 (70, 714)	388 (47, 648)	114 (34, 411)	0.003
Serum 25-hydroxyvitamin D (ng/dL)	20 (12, 25)	21 (14, 26)	-	0.86
Vitamin D <20 ng/mL, n (%)	25/52 (48)	16/34 (47%)	-	0.99
Parathyroid hormone	70 (51, 87) N=52	40 (29, 53) N=34	-	<0.001
Diabetes, n (%)	4 (7%)	1 (2%)	6 (7%)	0.47
Current cigarette use, n (%)	12 (21 %)	14 (33%)	5 (5%)	<0.001
SSRI use, n (%)	16 (28%)	8 (17%)	29 (32%)	0.16
Proton pump inhibitor use, n (%)	4 (7%)	2 (3%)	3 (3%)	0.62
Inhaled steroid use, n (%)	0 (0%)	0 (0%)	4 (4%)	0.15
Physical activity (MET-min/wk)	420 (162, 795)	600 (220, 960)	540 (250, 1020)	0.45
Menses per year	10 (5, 12)	11 (6, 12)	12 (9, 12)	0.21
	N=42	N=25	N=73	

Data are presented as mean (SD), median (25^th^, 75^th^ percentiles) or n (%)

Group comparisons by analysis of variance, chi-square or Fisher’s exact tests.

MBS = metabolic and bariatric surgery; RYGB = Roux-en-Y gastric bypass; VSG = vertical sleeve gastrectomy; SSRI = selective serotonin reuptake inhibitor

**Table 2.** Unadjusted mean DXA bone mineral density (BMD) by group.

	Group		*P*-value		
	RYGB	VSG	Controls	*Among groups*	*RYGB vs. VSG*
***BMD (g/cm*** ^***2***^ ***)***					
Whole body	1.106 (0.115) N=53	1.121 (0.088) N=43	1.126 (0.094) N=85	0.51	0.42
Lumbar spine	1.118 (0.124) N=55	1.123 (0.090) N=46	1.116 (0.117) N=86	0.95	0.73
Total hip	1.018 (0.135) N=50	1.034 (0.138) N=45	1.108 (0.120) N=89	<0.001	0.81
Femoral neck	0.929 (0.155) N=50	0.948 (0.137) N=45	0.998 (0.145) N=89	0.02	0.45
1/3^rd^ radius	0.749 (0.056) N=54	0.742 (0.062) N=48	0.741 (0.074) N=89	0.76	0.52
Ultra-distal radius	0.473 (0.066) N=55	0.475 (0.064) N=47	0.505 (0.070) N=90	0.008	0.88
***BMD Z-score***					
Whole body	−0.5 (1.1)* N=53	−0.4 (0.9)* N=43	−0.2 (0.8)* N=85	0.12	0.45
Lumbar spine	0.3 (1.0) * N=55	0.4 (0.8) * N=46	0.4 (1.0) * N=86	0.87	0.80
Total hip	0.3 (0.9) * N=50	0.3 (1.0) N=45	1.0 (0.8) * N=89	<0.001	0.85
Femoral neck	0.3 (1.1) N=50	0.4 (1.0) * N=45	1.0 (1.0) * N=89	<0.001	0.62
1/3 radius	0.5 (1.1) * N=54	0.1 (1.2) N=48	0.4 (1.0) * N=89	0.11	0.07
Ultra-distal radius	0.2 (1.0) N=55	0.0 (1.2) N=47	0.7 (1.0) * N=90	<0.001	0.30

RYGB = Roux-en-Y gastric bypass; VSG = vertical sleeve gastrectomy

Data are presented as mean ± SD.

Group comparisons by analysis of variance.

* Mean BMD Z-score is statistically different from 0, *p* < 0.05
